# Reproduction of Bone

**Published:** 1886-06

**Authors:** Joseph Kurtz

**Affiliations:** Professor of Clinical Surgery in the Medical College of the University of Southern California. Read before the Los Angeles County Medical Society.


ARTICLE III.

### REPRODUCTION OF BONE.

BY JOSEPH KURTZ. M. D.

(Professor of Clinical Surgery in the Medical College of the University of Southern California. Read before the Los Angeles County Medical Society.)

The term "reproduction" (or regeneration) of tissue may be used to represent a physiological, as well as a pathological, process. In the first sense it implies a new formation of a part, which was lost as the result of a perfect normal action, like the shedding and regeneration of epidermic and epithelial cells, the eyelashes (which completely change every 100–150 days), or the reproduction of hair or nails after trimming them. In the second sense, we understand the new formation of such parts which have been removed by an operation, performed to relieve either disease or injury.

As I intend to consider only the reproduction of bone tissue, it is not necessary to say any more about the first class of regeneration, as my subject will deal only with pathological cases; and in this respect man has not been as favorable endowed by nature as the lower region of the animal kingdom. Here we see that reproduction is nothing strange: spiders regenerate legs, some fishes their lost fins, and some lizards their extremities, the whole inferior maxilla the tail and even the eyes. But poor *homo*, who stands as perfection in the scale of creation, cannot boast of anything like it, his power of reproduction is very limited. Unfortunately, we see no new legs grow after an amputation, nor eyes after enucleation, and we are generally quite satisfied if we obtain a reasonable repair of a diseased or injured part.

It is stated that bone tissue is distinguished by a high degree of power of reproduction, but if we look for the new formation of an exact counterpart of the bone lost, whether by disease or injury, we are usually disappointed ; we rarely find anything better than a substitute, which, perhaps, resembles the original but little.

The repair of a fracture is generally accepted as a perfect regenerative process; but, if we come to consider this process thoroughly, I think the term "reproduction" is hardly applicable to it. The effect of a fracture is always an inflammation, followed by an exudation of a plastic material, which results in the formation of callus: first, the external callus, derived principally from the periosteum—the inflamed soft parts, cellular tissue, muscles and tendons furnishing but a very small amount; second, the internal callus, derived from the medulla, which forms a sort of a uniting wedge, and later on, after about thirty or forty days, an intermediary callus is formed at the ends of the fragments which finally completes their union. Still later, the superfluous external callus is absorbed and smoothed down, the medullary callus disappears, and gradually the bone assumes more or less the shape of the normal type. It may also gain the full strength it possessed before the injury, but if you examine the place of union very carefully you will find marked differences between it and the rest of the bone: it is always of a more compact consistence. It is the result of an inflammatory action, the same as we see in the repair of a wound in the soft parts, and I think the term "cicatrix" would be more applicable here than "reproduction."

I will now turn your attention to the process of restoration of bone which has been affected with caries. After a thorough removal of all detritus and other septic influences the cavity begins gradually to fill up with osteo-plastic material in the same manner as the ulcer of the soft tissues fills up with granulations, either of these being the result of an inflammatory exudation. If the cavity of the carious bone has been large and superficial, we rarely find that the bone will be covered with periosteum, but the cicatricial tissue of the soft parts will closely adhere to and so cover the bone. I think you will agree with me in this instance that the term "reproduction" is by no means the most suitable, and that the filling up of the cavity is again simply the result of a plastic inflammation. In caries of teeth no repair whatsoever takes place.

We shall now consider necrosis, a disease of bone, in connection with which the term "reproduction" (or regeneration) is more used than in the other conditions; in fact, new bony tissue must supply the place of the dead bone removed, if the patient should not be crippled for life. I think it best to begin here with a short description of the disease and see how the degeneration of the bone is brought about. Whether the necrosis be the result of an injury or of a periostitis or myelitis, due to syphilis, scrofula or tubercles, there always exists a sequestrating inflammation: the periosteum, being converted into a pyogenic membrane, is separated from the bone, while a fungating ostitis is set up in the bone, which forms the line of demarcation. The bone itself dies from a want of nutrition, and gradually a sequestrum forms which, when removed, leaves a cavity, small or large according to the size of the sequestrum, or also to the length of time of the disease. Generally at the time of removal of the dead bone we find that the periosteum has already done a good deal of work towards forming new bony tissue—indeed, by the time the sequestrum becomes completely detached we should expect a fairly formed involucrum, which may be hard enough to require the chisel and mallet, or the saw, to allow the extraction of the dead part. After the extraction the cavity left will soon be filled by an osteo-plastic material; again, the result of an inflammatory process, similar to the filling up of the cavity, in a carious bone, or, also, like the filling of an abscess cavity in the soft parts. This new formation of bony tissue does not exactly correspond to the size of the extracted bone, but frequently the substitute exceeds this in size and weight three or four times, and remains so. The most extensive osteo-plastic development we observe sometimes is central necrosis, and occasionally in cases in which the size of the sequestrum itself is but very small. Not rarely we meet with a very large involucrum of ivory hardness, to cut which may be very difficult, even with chisel and hammer, etc., to extract, perhaps, a sequestrum no larger than a small phalanx.

Not long ago I removed a piece of dead bone, from the femur of a man, scarcely an inch long and half an inch thick, while the bone with the involucrum seemed to me about three times as big as the femur of the other leg. Only a few days ago I operated on a man at the Sisters' Hospital before the class of students. I found a very thick tibia with a fistula running into it; the probe met rough bone, but no detached sequestrum. The man had been operated on before for the removal of dead bone at the Bellevue Hospital, N. Y. Though I could feel no detached bone, I felt sure I would find a large sequestrum; but, after enlarging the opening into the bone with chisel and hammer, I found nothing but a large bone abscess with a good deal of small detritus. This shows to you that new formation and defect do not always correspond. Again, we may find occasionally cases of partial or superficial necrosis, in which no involucrum forms at all, and in which hardly any osteoplastic material appears. Such cases often heal like superficial caries, the soft parts cicatrixing over it and leaving a depression in the bone. It is not very seldom either that reproduction of bone remains defective in cases of total necrosis of a diaphysis with solution of continuity, and that such cases may result either in shortening of the bone or in a false joint. As the periosteum is principally concerned in the production of new bony elements we cannot expect sufficient reproduction of bone in any case where the necrosed bone is not surrounded by periosteum. These facts seem to me conclusive that reproduction or regeneration of bone in the true sense of the word does not take place in necrosis either, but that here also we find that the defect is replaced by an osteo-plastic material, the result of a plastic inflammation again.

It is usually understood that joints are reproduced after exsection, and no doubt very favorable results are obtained as regards the usefulness of the limb after such operations. I have made quite a number of these operations, and confess that I felt very happy over my little success, and I take pleasure in showing to you here a few of the bones which I removed. This first one you will readily recognize as the head of a femur of a child. The patient was a boy, aged nine years, suffering from coxalgia in the third stage; he made an excellent recovery, and now runs over the plains of San Fernando perhaps a good deal faster than any of us here could, in spite of over an inch shortening. The second specimen here is, as you see, the head and part of the shaft of a humerus, taken from a man aged 45, who had been shot through the arm by Indians. The last time I heard from him he stated that he could pitch hay almost as good as before he was injured, but his arm was considerably shortened, There is a third specimen that is worth looking at, it is quite a large part of the inferior maxilla. The patient, from whom I removed this, had his jaw crushed by means of brass knuckles, which were applied to him by one of his friends. A very short time after removing this bone I had occasion to witness this patient chew a beefsteak, which was not by any means of the most tender kind.

Many more specimens I could produce here, but I am afraid I have taxed your patience too much already; but I may mention that most of those were exsected with good, and a few with bad, results. I have also seen many cases of exsections of joints of other surgeons, with various results, but neither these nor my own cases have ever convinced me that a joint of exactly the original type was reproduced. I have always endeavored, as much as practicable, to make my operations subperiosteal ones, and conducted the after-treatment with appliances so as to avoid deformity as much as possible, and in spite of all precautions the limbs were shortened and the jaws were slightly deformed. I believe the epiphyses are rarely, if ever reproduced. In exsections of the kness and elbow-joints the results are not always very flattering; total ankylosis of the knee is frequent, and partial ankylosis of the elbow must often satisfy our ambition. At best we may obtain a pseudarthrosis, which may answer well enough, but I doubt whether you may ever find the condyles of the femur or the humerus regenerated. Neither is the new-formed cartilage the same as the normal cartilage of a joint; it has not the same brilliant white appearance, nor has its surface the exact shape of the lost one. It consists rather of a fibrous tissue interspersed with some hyaloid cartilaginous substance, shaped by the grinding of the bone-ends upon each other.

The favorable results obtained from exsections of joints are really not due to the reproduction of articulating bone-ends as much as to the preservation of the insertions of the muscles or tendons, Exsections of the elbow joint give probably the best results among all the operations on joints. The reason for this is obvious, this joint being so superficial we have very easy access to it, and are generally enabled to remove the bones without interfering much with the soft tissues. Here we have the best possible chance to make a perfect subperiosteal operation, and neither extensor-, flexor-, pronator- nor supinator-muscles need to be severed. If the after-treatment is fairly conducted, and passive motions not neglected, we may have good reason to expect a movable joint with about as much usefulness as the original one. But with all it will only be a pseudarthrosis, or a false joint similar to the one formed by an ununited fracture.

As I have already stretched this paper longer than is wholesome for your patience, I must conclude without considering the result of such operations on any more joints; but I believe I have sufficiently demonstrated to you the fact that reproduction of a fac simile of the removed bone or joint is but very rarely, if ever, obtained, and that the healing process in the soft tissues is the result of a plastic inflammation.—*Southern California Practitioner.*

